# Is reduced female survival after mating a by-product of male-male competition in the dung fly *Sepsis cynipsea*?

**DOI:** 10.1186/1471-2148-7-194

**Published:** 2007-10-17

**Authors:** Y Teuschl, DJ Hosken, WU Blanckenhorn

**Affiliations:** 1 Zoologisches Museum, Universität Zürich, Winterthurerstrasse 190, CH-8057 Zürich, Switzerland; 2 Centre for Ecology & Conservation, School of Biosciences, University of Exeter, Cornwall Campus, Tremough, Penryn, Cornwall, TR10 9EZ, UK

## Abstract

**Background:**

In a number of species males damage females during copulation, but the reasons for this remain unclear. It may be that males are trying to manipulate female mating behaviour or their life histories. Alternatively, damage may be a side-effect of male-male competition. In the black scavenger or dung fly *Sepsis cynipsea *(Diptera: Sepsidae) mating reduces female survival, apparently because males wound females during copulation. However, this damage does not seem to relate to attempted manipulation of female reproduction by males. Here we tested the hypothesis that harming females during mating is an incidental by-product of characters favoured during pre-copulatory male-male competition. We assessed whether males and their sons vary genetically in their ability to obtain matings and harm females, and whether more successful males were also more damaging. We did this by ranking males' mating success in paired competitions across several females whose longevity under starvation was subsequently measured.

**Results:**

As previously reported, our results show mating is costly for female *S. cynipsea*. However, variance in female longevity was not explained by male identity, family, body size, number of previous copulations, or copulation duration. Nevertheless, there was a positive correlation between the harm fathers inflicted on their mates (affecting female longevity) and the harm sons inflicted on theirs. Additionally, family identity significantly influenced male copulation success.

**Conclusion:**

Our results indicate a heritable component of some yet unspecified male trait(s) that influence harm and mating success. However, there was no relationship between copulation success of fathers or sons and the mean longevity of their mates. We therefore found no support for harm being a side effect of traits favoured in pre-copulatory male-male competition.

## Background

Males and females frequently have different optima for many aspects of reproduction including parental investment and mating frequency [1-6]. Reproduction is therefore potentially loaded with conflict (reviewed in [7-11]), and while conflicts are proceeding, there will be selection for manipulative abilities that allow one or the other sex to achieve their respective optima [12-14]. There are a number of ways to be manipulative, and all involve some cost to the manipulated party as they are moved away from their reproductive optima [13,15], a situation recently termed conflict load [13].

In addition to conflict load, manipulative traits can impose further costs, termed harm, and these can be partly distinguished from conflict load by the fact that harm frequently seems to negatively impact on the fitness of the manipulating and the manipulated sex [13]. It is largely this fact that makes harm so difficult to understand (but see [16]), but an increasing number of studies document it. Perhaps the clearest cases involve males damaging females during copulation. For example, in the beetle *Callosobruchus maculatus *the male intromittent organ physically damages females, reducing female life-span [17] (but see [18]), and the traumatic insemination that occurs in the bed bug *Cimex lectularius *can also decrease female longevity [19]. Similarly, seminal fluids have been shown to negatively impact on female lifespan in *Drosophila melanogaster *[20], with sex-peptide recently being implicated in this phenomenon [21,22].

Two general hypotheses have been proposed to explain the evolution of harm [13]. The adaptive harm hypothesis suggests that harm occurs because males are directly trying to manipulate female behaviour. This could occur if harm reduces the likelihood that females mate again, or prolongs remating intervals, because if either occurs, males increase the likelihood that females use their sperm [23]. Furthermore, males may be trying to manipulate female oviposition rates [24]. In this case, harm causes females to increase their reproductive output in a manner analogous to terminal investment. Models suggest that both of these strategies could in principle work [23,24], but experimental tests of these ideas found no evidence to support them [25-27]. An alternative to adaptive harm is that harm is a side-effect of traits favoured by male-male competition [13,28], and there is some evidence for this in *Drosophila*, where genotypes most successful in sperm competition are more damaging to females [29]. Analogous results are found in the cockroach *Nauphoeta cineria*, where male-male competition selects for males that reduce female fitness [30,31].

The black scavenger or dung fly *Sepsis cynipsea *(Diptera: Sepsidae) has also been extensively studied in this context. Females of this species show very conspicuous resistance behaviour to mating [32-34]. As soon as females arrive at dung pats for egg laying, they are mounted and guarded by males, and copulation only occurs after females have laid their eggs. Females that are unwilling to mate shake vigorously and extensively from side to side, bending their abdomen downwards until males leave [32], and only about 40% of the pairs that form in the field eventually copulate [32,33,35]. Males seem unable to force copulations [[36-38]; cf. [39]]. Female reluctance to mate in this species is seen as a classic example of conflict over mating [25,34,36,37,40,41] and has been interpreted to result from high mating costs for females [33,36,40]. In support of this interpretation, it has been shown that mated females have more wing injuries and die sooner than unmated females, with mortality apparently elevated by injuries to the female reproductive tract caused by male genitalia during copulation [42]; but compare [43] for another sepsid species). However, males do not appear to be benefiting from this harm by manipulating female oviposition or mating, because although injuries seem to increase with copulation number [42], females do not lower their remating rate or increase reproductive investment with increasing harm [25]. Post-oviposition copulations, which are generally rare but typical for sepsid flies [32,44], make advantages of injuring females during mating even less plausible: from a male perspective, females have to survive until they lay their next clutch because only then will females use the damaging male's sperm, but harming females reduces the likelihood they will survive to this point. Nevertheless, while we have no evidence for adaptive male harm, the possibility remains that harm represents collateral damage.

In this study we test the idea that injuring females during mating is a by-product of traits augmenting male reproductive success in pre-copulatory competition with other males. For example, extremely vigorous males may be better at securing copulations, but may also be rougher during copulation, analogous to the situation found in *D. melanogaster *[29]. In a laboratory experiment we assessed whether males differ in their ability to harm their mates, and compared fathers with their sons to estimate the heritability of this trait. At the same time we investigated whether or not male (fathers' and their sons') mating success, when in competition with other males, is positively correlated to female damage. Since we evaluated these questions at the family mean level, we also assessed whether or not there is additive genetic variation for these attributes [45-47].

## Results

### Influence of reproduction on female residual longevity

**Figure 1:**
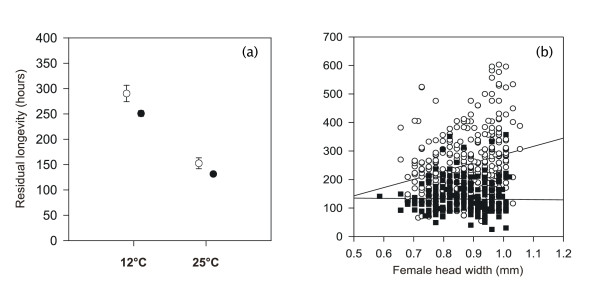
Effects of (a) copulation (white circles no copulation; black circles one copulation) on (square-root-transformed) female residual longevity at 12°C and 25°C (in h) and (b) body size on female residual longevity at 12°C (white circles) and 25°C (black circles) for copulated females only (son experiment).

Before investigating specific male effects, we first investigated the effects of factors other than male characters (son experiment only). Females that copulated died earlier than control females of similar age that were not allowed to copulate (ANCOVA with copulation and temperature treatment as factors and body size as covariate: *F*_1,725 _= 9.24, *p *= 0.002; Fig. 1a; cf. [41]). Flies of the 25°C treatment died sooner (*F*_1,725 _= 5.01; *p *= 0.024; Fig. 1a). Larger females survived starvation for longer (*F*_1,725 _= 11.57; *p *= 0.001), but only at 12°C (temperature by body size interaction: *F*_1,725 _= 20.02; *p *< 0.001; Fig. 1b).

To specifically investigate the influence of egg laying on survival (as opposed to copulation plus egg laying, which were not differentiated in the previous analysis), 25°C flies were analyzed separately (because in the 12°C treatment females were not allowed to lay) in an ANCOVA with copulation as a factor and the number of clutches laid and female size as covariates. The number of clutches a female laid in the son experiment did not influence her residual longevity (*F*_1,310 _= 1.28; *p *= 0.218). Similarly, egg number did not influence the residual longevity of the females mated with the fathers (Table 1).

**Table 1:** ANCOVA of the factors influencing individual (left) and family mean (right) (square-root-transformed) residual longevity of females mated with fathers (all non-significant interactions removed from the model)

Residual longevity					Mean residual longevity				
	*df*	*MS*	*F*	*P*		*df*	*MS*	*F*	*P*
			
Egg number	1	0.084	0.017	0.896	Mean egg number	1	1.182	1.072	0.306
Female size	1	0.043	0.009	0.926	Mean female size	1	2.636	2.392	0.129
Copulation number	1	4.758	0.965	0.327					
Male identity	56	4.511	0.915	0.644	Male size	1	0.735	0.667	0.418
Error	173	4.932			Error	46	1.102		

### Influence of male identity on female residual longevity

The residual longevity of females mated to the fathers was not influenced by male identity, the number of copulations a male previously had, female size or the total number of eggs females laid (Table 1). Residual longevity of females mated with sons was also tested, and here we included the fathers' identity as a random factor. Neither fathers' nor sons' identity had significant effects (Table 2).

**Table 2:** ANCOVA of the factors influencing individual (left) and family mean (right) (square-root-transformed) residual longevity of females mated with sons (all non-significant interactions removed from the model)

Residual longevity	*df*	*MS*	*F*	*P*	Mean residual longevity	*df*	*MS*	*F*	*P*
Temperature	1	722.107	106.549	< 0.001	Temperature	1	18.856	7.311	0.010
Son ID (father ID)	21	7.762	1.145	0.307	Male size	1	0.000	0.000	0.989
Father ID	17	6.044	0.782	0.695	Father ID	14	4.494	1.742	0.088
Female size	1	15.007	2.214	0.139	Mean female size	1	1.173	0.455	0.504
Copulation duration	1	3.685	0.544	0.462	Mean copulation duration	1	2.226	0.863	0.359
Copulation number	1	0.027	0.004	0.949					
					Temperature × mean copulation duration	1	17.693	6.860	0.013
					Temperature × mean female size	1	17.406	6.749	0.013
					Male size × father ID	14	4.519	1.752	0.086
					Temperature × mean copulation duration × mean female size	2	8.712	3.378	0.045
Error	162	6.777			Error	37	2.579		

To include male body size instead of male identity in an alternative analysis, the average female residual longevity of all his mating partners was calculated for each individual male (Tables 1 and 2 for fathers and sons, respectively). As in the analysis before, none of the variables, including male size, influenced residual longevity of females mated with the fathers (Table 1). Male size also did not influence the mean residual longevity of females mated with the sons (Table 2). There was a trend, apparently depending on male body size, for the residual longevity of females that mated with brothers to be similar (father identity effect and father identity by male size interaction in Table 2). As in the previous analyses, females mated with sons lived longer in the 12°C treatment and when they were larger (temperature effect and temperature by mean female size interaction in Table 2; cf. above). Furthermore, females lived longer when they copulated for longer, but only at 12°C (temperature by mean copulation duration and temperature by mean copulation duration by female size interactions in Table 2). This is probably mediated by the fact that males copulate longer with larger females, and that large females live longer in the 12°C treatment (cf. above).

**Figure 2:**
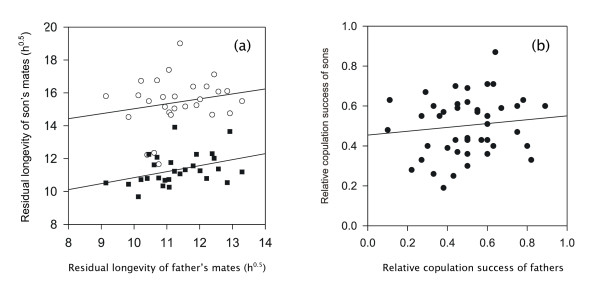
Family-mean regressions of (a) (square-root-transformed) residual longevity (in h) of fathers' mates on that of their sons' mates at 12°C (white circles) and 25°C (black squares), and of (b) the cumulative copulation success of fathers on the cumulative copulation success of their sons (at 25°C).

To investigate a possible heritability of harming females, mean residual longevity of all the mates of fathers and those of sons was compared in a parent-offspring regression including temperature treatment as a factor. There was a marginal overall relationship (*F*_1,55 _= 3.95; *p *= 0.052; Fig. 2a), caused by a strong and significant correlation in the 25°C treatment (*r *= 0.382; heritability *h*^2 ^= 0.73 ± 0.35 (SE)), while the association was weaker in the 12°C treatment (*r *= 0.199; *h*^2 ^= 0.60 ± 0.58).

### Are some genotypes more successful in acquiring matings than others?

In 200 of 370 (54.9 %) of the male pairs among fathers and 383 of 724 (52.9 %) of the male pairs among sons the red male was scored as winner, so the artificial colouring of the wings did not appear to influence a male's ability to secure copulations (binomial test for fathers: *p *= 0.132; for sons: *p *= 0.128). As shown before [36], in direct competition in the laboratory the larger of two males is more likely to obtain copulations (binary logistic regression with the size difference between the interacting males as the dependent variable; for fathers: *p *= 0.017, *n *= 230; marginal effect for sons: *p *= 0.062, *n *= 615).

To determine if male copulation success was similar for half brothers, the cumulative copulation success of each son (= proportion of encounters won against all competitors) was analyzed using ANCOVA with the father's identity as a random factor and the son's size as a covariate. The father's identity (i.e. family; *F*_46,125 _= 1.58, *p *= 0.036) significantly affected copulation success, suggesting a heritable component. However, at the same time cumulative copulation success of fathers and that of their sons was not significantly positively correlated (*r *= 0.132, *n *= 47, *p *= 0.377; Fig. 2b). Moreover, body size in this experiment also showed no heritable component in either a half-sib ANOVA design with father as random factor (*F*_49,147 _= 1.06; *p *= 0.399) or a father-son correlation (*r *= -0.058, *n *= 40, *p *= 0.721).

### Do mates of more successful males have reduced residual longevity?

**Figure 3:**
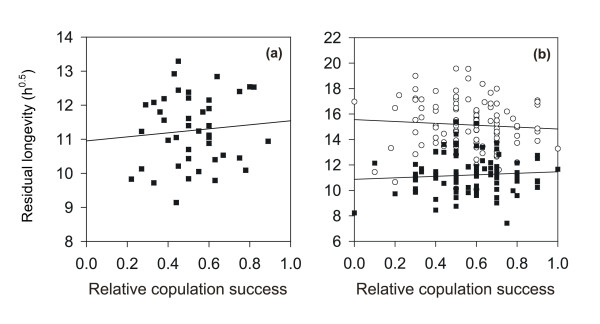
Regressions of the family (square-root-transformed) mean residual longevity (in h) of their mates on the cumulative copulation success of (a) fathers (at 25°C) and (b) sons at 12°C (white circles) and 25°C (black squares).

For fathers there was no relationship between their cumulative copulation success and the mean (square-root-transformed) residual longevity of their mates (*r *= 0.062, *n *= 43, *p *= 0.694; Fig. 3a), nor was there such a relationship for the sons in the 12°C (*r *= -0.118, *n *= 112, *p *= 0.215) or the 25°C treatment (*r *= 0.073, *n *= 104, *p *= 0.460; Fig. 3b). However, it may be that a male's ability to harm their mating partner diminishes with his number of mates as he becomes sperm depleted or otherwise weakened. To test this, we first checked whether female residual longevity changed (i.e. here specifically increased) with her rank order as a mate of a particular male (= random effect); there was no such relationship for either fathers or sons at any of the two temperatures (*r *< 0.1, *p *> 0.3). Furthermore, analogous to the first analysis above (Fig. 3), we further regressed a male's cumulative copulation success on the residual longevity of only his first mate, assuming his ability to harm females would be strongest and hence most apparent then. Again, there was no such relationship for either fathers or sons at any of the two temperatures (*r *< 0.1, *p *> 0.4).

## Discussion

We did not find any support for the hypothesis that harming females is associated with success in pre-copulatory male-male competition in *Sepsis cynipsea*. We found some evidence for the heritability of both a male's ability to harm his mates (here measured as female residual longevity at two temperatures; Fig. 2a) and male mating success (here measured as the total number of copulations obtained). However, females that mated with more successful male genotypes did not have reduced residual longevity (Fig. 3), as would be expected if harm was a collateral effect of pre-copulatory male-male competition [29-31]. It is also unclear why the heritability of harm was quite high (Fig. 2a), but at the same time not repeatable across females of a given male. Nevertheless, in agreement with previous work, which found a negative association between copulation number and female longevity [42], mated females died sooner than unmated females, and this effect was largely independent of the number of clutches laid, i.e. female costs of reproduction. This contrasts markedly with results for a related sepsid fly (*Saltella sphondylii*), where copulation had no detectable effect on female longevity while egg laying did [48].

We also found (for sons) that females in the 25°C died sooner than those of the 12°C treatment (Fig. 1a). The main difference between these two treatments was that the former females lived for one week alone at 25°C, where they could eat and oviposit, before they were moved to 12°C. Their shorter residual longevity probably reflects a cost of reproduction and/or the added physiological demands of living at 25°C. Interestingly, female size influenced residual longevity only in the 12°C treatment, where females were simply starved and could not reproduce, reflecting the relatively more efficient metabolism of larger individuals [49]. If females during their week in the 25°C treatment used most of their energy reserves to produce eggs, and if larger females utilize proportionally more energy for reproduction, this may explain the disappearance of the size relationship in the 25°C treatment. The relatively complex nature of these size-dependent effects on female residual longevity highlights the fact that we do not precisely know what makes mated females die more rapidly.

The 25°C treatment of the sons is directly comparable to that of the fathers, and indeed residual longevity of their mates was similar in absolute terms in both data sets (Fig. 2a), as would be expected if female mortality after mating is due at least in part to wounds inflicted by males [42]. However, female mortality was not influenced by male body size, male identity or copula duration. This differs from *Drosophila melanogaster*, where the preferred larger males harm and reduce female fitness more than smaller males [50,51]. In further contrast to *D. melanogaster*, where seminal fluid increases egg laying behaviour but shortens female lifespan [22,22], in *S. cynipsea *copulation does not appear to enhance egg laying [38] or be otherwise toxic for females, although in another study longer copulations did reduce the number of offspring a female produced [52]. Males in our experiment copulated multiply within a short time interval, which may influence the amount of ejaculate transferred to females. While we do not know how often males copulate in the field, or whether or not our males became ejaculate depleted here, the number of previous copulations a male had did not influence female residual longevity. Moreover, because particular males were not found to be generally more harmful, it does not seem that larger males are more damaging [cf. [42]].

Admittedly, in our experiment we might have missed the possibility that specific male genotypes are more harmful for specific female genotypes in a manner analogous to genetic incompatibility (e.g. [53,54]). For example, Nilsson and colleagues [55] showed that in the flour beetle *Tribolium castaneum *both female and male genotype influence female fitness in complex ways, with similar fitness interactions found in a range of taxa [56-58]. We cannot exclude such male-female interactions in *S. cynipsea*, as females copulating with the same male differed in their residual longevity. However, we cannot distinguish if this is merely random, purely female mediated or due to a female-male genotype interaction, because we did not vary female genotype (i.e. family) in the same manner as and independently from male genotype. Nevertheless, if levels of harm depend upon male-female interactions, the heritability of harm should be low, but it was not.

Our statistical support for the heritability of male harmfulness was not very strong overall, but our estimate is relatively high [cf. [59-62]]. Hoffmann [63] showed that heritability estimates for mating behaviour may be underestimated if they are based on single events; they can be much higher when several mating events are taken into account. This should not be a major problem here, as in our experiment heritability was estimated from at least four matings for each male and treatment, although the accuracy of estimates can still increase with the inclusion of up to six matings [63].

As previously shown in *S. cynipsea*, large males have a mating advantage in direct male-male competition [35,36]. However, in the current study this effect was only weak for sons. Our experimental design included several copulations within a short time, and large and otherwise superior males may have been exhausted and less successful in securing subsequent copulations. We have no data on the number of matings obtained by males in the field, although in nature males probably encounter more competitors, acquire fewer matings and are more stressed by additional environmental factors such as predators. In *S. cynipsea *the large male advantage is not primarily due to direct aggression between males. When both males were seen on top of the female and females were willing to copulate, the first male usually ended up as the winner; take-overs were recorded very infrequently. This suggests the winning males were generally the first ones to encounter and mount the female, which is comparable to previous results [36]. It is not clear if larger males are more active, faster, or if they constrain the activity of the smaller males in some way. However, virgin females tend to copulate with the first male encountered or not at all [38], and if larger males see, approach and mount females more readily, this would explain at least partly the large male advantage found in the field and the laboratory [33,35,36,64,65].

Body size in *S. cynipsea *is heritable [62,66], but highly phenotypically plastic, depending on temperature and the amount of larval food (dung) available [67]. Our failure to detect significant size heritability in this study may be because we did not control larval food levels. However, this was done on purpose as it allowed us to disentangle purely size-mediated effects and the genetic quality of males independent of size. On the other hand, larval food availability can influence adult condition and may therefore mask underlying genotypic effects to some degree. Despite this potential confound, we found significant family influences on mating success, although the non-significant father-son body size correlation suggests that some genetic male quality other than size underlies this.

## Conclusion

Overall, our study provided no evidence that harming females during copulation is a by-product of some other trait increasing a male's success in pre-copulatory male-male competition; at least there is no simple linear effect. Since previous studies also failed to identify advantages to males harming females [25,42], the reasons for male harm remain enigmatic in *S. cynipsea*. However, we only investigated pre-copulatory male-male competition. Harming females may correlate with some post-copulatory advantages such as sperm competitive ability [28-30]. To date we have little information on post-copulatory sexual selection in *S. cynipsea *(but see [68]), but if post-copulatory male-male competition were associated with harm levels, harmfulness could still be heritable, and we found some evidence for this. Alternatively, the greater mortality of mated females may be an inescapable cost of mating *per se *or of some associated physiological processes, independent of any male-female reproductive conflict. It would be helpful if we could identify the exact mechanism increasing female mortality after mating in this species. To date our data suggest that harm persists not because of associations between harming females and advantages in male-male or male-female interactions, but because it is a pleiotropic (i.e. non-adaptive) effect of mating itself. One other possibility is that harm persists because interactions between male-female genotypes generate variability in the damage inflicted, rendering selection against single male genotypes ineffective. All of these questions will be the subject of future investigations.

## Methods

Flies used in this experiment stemmed from a laboratory population kept at standard conditions with sugar, pollen, water and dung at 25°C, 60% relative humidity and 12 h photoperiod. The population was initially started with 120 flies collected on two days in May 2002 in Fehraltorf, near Zürich, Switzerland. To generate virgins for experiments, individuals were sexed upon emergence and housed in single-sex group containers.

To test whether or not some males inflict more damage to females than others and if male harm is heritable, we compared the survival of females that copulated with the same males, and we compared their survival to the survival of females that copulated with the sons of these males. Additionally we compared the copulation success of fathers and sons.

### Treatment of fathers

To distinguish competing males, the wings of 60 males were coloured randomly either with red or green colour, so that half of the males were red and the other half were green. In each trial, up to 15 random pairs of males were transferred into a 50 ml test vial (2 males/vial). To each of these pairs a virgin female aged of 3 – 5 days (when they are most receptive: [38]) was added and the three flies were observed. If females shook vigorously for approximately 10 min they were exchanged for other females. Longer persistence of males does not significantly increase their probability of copulating [36], and 80% of all copulations occur within the first 10 min after the first male mating attempt. Furthermore, shaking indicates female unwillingness to mate, more so than assessment of male quality [36]. Indeed, if the females were shaking vigorously for some time, they usually did so with both males. Females were exchanged up to 5 times until one of the two males obtained a copulation; if by then no copulations had occurred, the pair was not scored. The copulating male was scored as the winner of a pair. After all pairs were scored, males were rotated to obtain new (random) combinations of red-green male pairs, and the process was repeated using different females until each male was tested in 10 different trials against 10 different males. These trials were spread over 2–4 days. Only males with more than six completed trials were included in the final analysis.

### Treatment of mothers

After copulation females were held alone in 100 ml vials with sugar, pollen and water and kept at 25°C and 60% relative humidity for 10 days. A maximum of 5 females that copulated with a given male were kept. To obtain 5 mates for each male, those males that did not achieve 5 copulations were given the possibility to copulate with females without a competitor. Females were provided thrice with fresh cow dung for egg laying over 1 week. To generate offspring for the son experiment, eggs were counted and each dung portion with eggs was transferred into plastic containers and kept at 25°C (offspring are not produced or take forever to emerge at 12°C). Offspring were counted at emergence and sons were held separated by family to test them in a manner similar to their fathers (as described below).

To estimate the magnitude of harm to females, after their third egg laying opportunity females were transferred into 50 ml glass vials containing only water, and moved to a 12°C climate chamber, where they were checked three times per day for death. At colder temperatures physiological processes slow down and females survive longer, producing greater variation in longevity. This treatment served to enhance the likelihood of detecting mortality differences inflicted by individual males, especially because stress is frequently required for experimental effects to become manifest [69,70], as shown previously for this species [41]. In what follows, the survivorship in the 12°C climate chamber under complete starvation is referred to as residual longevity, but this measure is equivalent to the term starvation resistance used in the *Drosophila *literature [71].

### Treatment of sons

Three sons from different mothers (i.e. half brothers) were tested in exactly the same way as their fathers (described above). All mates of these males were kept and split randomly into two groups: a 12°C and a 25°C treatment. 12°C females were transferred immediately after copulation into vials containing water and moved into a 12°C climate chamber; 25°C females were given the possibility to lay eggs thrice, during which time they were kept at 25°C with sugar, pollen and water. Eggs were not counted; we only checked whether or not a female laid. After 1 week these females were also moved to the 12°C climate chamber with only water. We did this because the mating wounds females incur during copulation may only have a negative effect after egg laying into cow dung. Additionally, this treatment was directly comparable to the one used for the fathers' mates described above. Again, we checked three times per day for death. As a control, 2 × 50 unmated females of approximately the same age were chosen randomly from the female group container and subjected to the 12°C and 25°C treatments.

### Statistical analyses

Head width of all males, their mates and the control females was measured under a dissecting microscope at 40× magnification and included in all analyses as a covariate. Head width is a good estimate of body size in this species [66].

Residual longevity (after copulation) was square-root transformed and outliers were excluded (i.e. data points more than 3 standard errors away from the mean), as they may indicate inadvertent access to nutrients. Only copulation durations longer than 10 min and shorter than 60 minutes were included. In statistical analyses including male identity, only males that copulated with at least three females were included. In the analysis of the sons only males mated to at least two females in each temperature treatment were included. In analyses including fathers and sons only fathers with data for more than two sons were included.

## Competing interests

The author(s) declares that there are no competing interests.

## Authors' contributions

All three authors contributed to the design of the study and participated equally in the execution of the experiments. YT wrote the initial manuscript as part of her Ph.D. thesis, and DJH and WUB contributed substantially in writing and re-writing. YT performed the statistical analyses with assistance from WUB. All authors have read and approved the final version of this manuscript.
